# The decline in paediatric malaria admissions on the coast of Kenya

**DOI:** 10.1186/1475-2875-6-151

**Published:** 2007-11-15

**Authors:** Emelda A Okiro, Simon I Hay, Priscilla W Gikandi, Shahnaaz K Sharif, Abdisalan M Noor, Norbert Peshu, Kevin Marsh, Robert W Snow

**Affiliations:** 1 Malaria Public Health & Epidemiology Group, Centre for Geographic Medicine Research – Coast, Kenya Medical Research Institute/Wellcome Trust Research Programme, P.O. Box 43640, 00100 GPO, Nairobi, Kenya; 2 Spatial Ecology and Epidemiology Group, Department of Zoology, University of Oxford, Tinbergen Building, South Parks Road, Oxford, OX1 3PS, UK; 3 Ministry of Health, Afya House, Cathedral Road, P.O. Box 30016, 00100 GPO, Nairobi, Kenya; 4 Centre for Geographic Medicine – Coast, Kenya Medical Research Institute, P.O. Box 230, Kilifi, Kenya; 5 Centre for Tropical Medicine, University of Oxford, John Radcliffe Hospital, Headington, Oxford, OX3 9DU, UK

## Abstract

**Background:**

There is only limited information on the health impact of expanded coverage of malaria control and preventative strategies in Africa.

**Methods:**

Paediatric admission data were assembled over 8.25 years from three District Hospitals; Kilifi, Msambweni and Malindi, situated along the Kenyan Coast. Trends in monthly malaria admissions between January 1999 and March 2007 were analysed using several time-series models that adjusted for monthly non-malaria admission rates and the seasonality and trends in rainfall.

**Results:**

Since January 1999 paediatric malaria admissions have significantly declined at all hospitals. This trend was observed against a background of rising or constant non-malaria admissions and unaffected by long-term rainfall throughout the surveillance period. By March 2007 the estimated proportional decline in malaria cases was 63% in Kilifi, 53% in Kwale and 28% in Malindi. Time-series models strongly suggest that the observed decline in malaria admissions was a result of malaria-specific control efforts in the hospital catchment areas.

**Conclusion:**

This study provides evidence of a changing disease burden on the Kenyan coast and that the most parsimonious explanation is an expansion in the coverage of interventions such as the use of insecticide-treated nets and the availability of anti-malarial medicines. While specific attribution to intervention coverage cannot be computed what is clear is that this area of Kenya is experiencing a malaria epidemiological transition.

## Background

Since the inception of the Roll Back Malaria (RBM) movement in 1996 [1], billions of health dollars have been committed by the international donor community to reduce the burden of malaria in Africa [2], estimated to be over a million deaths directly due to *Plasmodium falciparum *annually [3]. However there are remarkably few documentations of changes in disease burden associated with increases in access and use of interventions funded by new international donor agency money.

In Kenya the expansion of coverage of both ITN and effective ACT therapy (artemether-lumefanthrine) has occurred very recently. Between 2004 and 2005 ITN coverage among children aged less than five years rose from 7% to 24% and by the end of 2006 had risen to 67% coverage [4]. Despite delays in implementing the revised drug policy supporting the use of AL [5], over 85% of rural clinics had AL in stock between August and December 2006 [6]. Paediatric admission time-series from three Kenyan hospitals were assembled to explore the impact of parallel changes in intervention coverage and malaria disease burden.

## Methods

### Paediatric admission data

**Figure 1:**
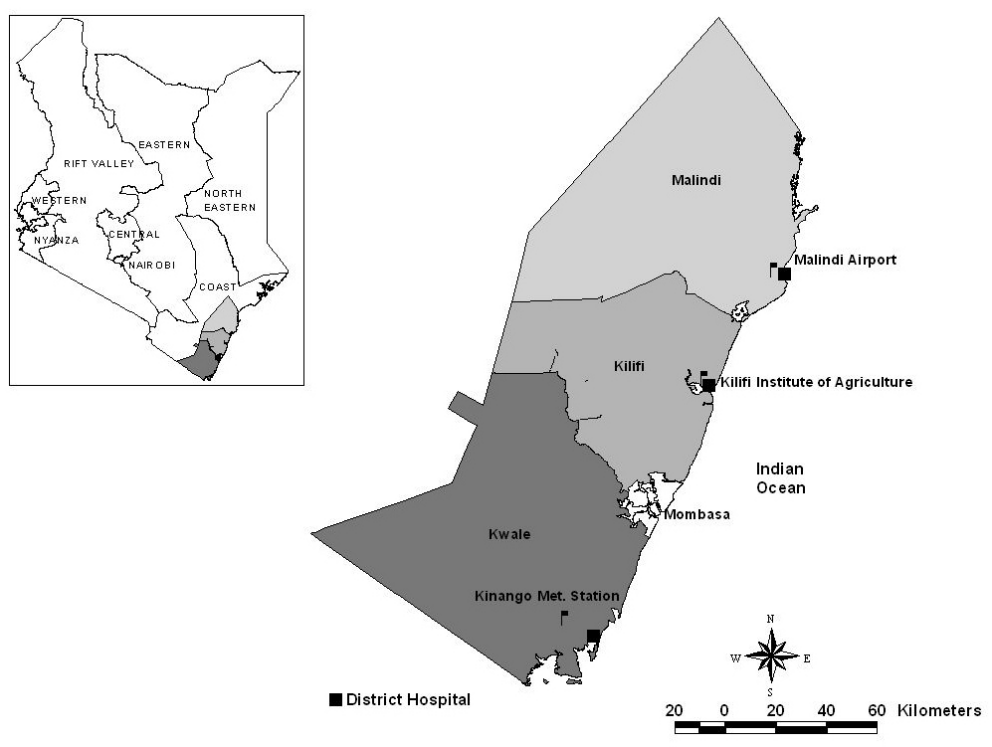
Map showing the three study districts and the location of the metrological station in relation to the hospital facility. Inset is a map of Kenya showing location of three districts.

Three district hospitals were selected purposively along a 170 km stretch of the Kenyan coast at Malindi, Kilifi and Msambweni (located in Kwale district). They are located in three different districts of Coast Province, and all serve populations who share similar climatic, ecological and economic characteristics (Figure 1).

Paediatric ward in-patient registers at Malindi and Msambweni were identified for all months from January 1999 to March 2007. These were arranged serially to check whether these represented a continuous, uninterrupted series. Each admission entry in the registers was recorded on a separate tally sheet indicating the month of admission, whether a primary working diagnosis of malaria had been defined for the child, whether the admission diagnosis was not malaria and whether the child had survived admission. Separately, hospital death certificates were reviewed for the same period to identify any paediatric deaths that may have not been recorded in the admission ward books. All paediatric admissions were assumed to be aged between birth and 15 years. Individual register entries were not reconciled with patient notes and thus we have assumed that the admission diagnosis remained the clinical management diagnosis and it is used here as the diagnosis of analysis. The reliability of slide confirmed malaria diagnosis at admission was not validated from hospital or laboratory records as these procedures are variously performed in most Kenyan district hospitals [7] and results are rarely used to refine a diagnosis [8-10].

At Kilifi district hospital a sophisticated paediatric ward surveillance system has been *in situ *since 1989 to form the basis of a series of clinical studies on the pathogenesis of malaria, pneumonia, malnutrition and neonatal illness [11-14]. Clinical and laboratory coverage is provided 24 hours each day throughout the year. Demographic details and clinical histories are recorded on every admission and a finger prick blood sample taken for malaria parasitology and basic haematology. Following a clinical examination at admission further clinical and laboratory investigations are undertaken as indicated. Clinical and laboratory findings, clinical progress and response to therapy were reviewed at discharge to derive a primary diagnosis and recorded on a standard admission proforma later entered onto a centralized database. Data for the present study were reassembled for the period January 1999 through to March 2007 and summarized by month as a discharge diagnosis of malaria or non-malaria for all admissions aged between birth and 15 years of age.

### Ancillary data

Seasonal patterns of malaria hospitalization are related to monthly rainfall precipitations along the Kenyan coast [15] and relate principally to the population dynamics of the dominant vector species [16]. Monthly rainfall data in decimal mm were obtained from meteorological offices located 3 km (Malindi), 2 km (Kilifi) and 25 km (Msambweni) from the respective district hospitals and were complete across the same time-period as the paediatric ward surveillance (Figure 1). The Kenyan coast is an area where sporogony in the vector population and hence malaria transmission, is not limited by ambient temperature and therefore temperature data were not included in the analysis.

Natural population growth over the 8.25 observation years is likely to have affected the size of population accessing these three hospitals. Precise rates of admission adjusted for monthly denominator size were not possible for the hospitals as the catchment for these services cannot be rigorously defined over time [17]. Non-malaria admissions data were also collected, however, to help calibrate for population and other issues affecting hospital usage.

### Data analysis

Analysis was undertaken using STATA version 9.2 (Statacorp 2003, College Station, USA). Hospital admission data obtained over the 99 months of surveillance between January 1999 and March 2007. Malaria admission case totals were assembled chronologically by admission month and non-malaria admissions similarly assessed for comparison purposes. They were examined with time series analysis that used malaria admission cases as the main outcome.

Smoothing techniques (moving averages) were used to filter short-term annual fluctuations and thus highlight longer-term trends present in the each of the data series. Malaria, non-malaria and rainfall, time series data from Kilifi, Kwale and Malindi were subject to 13-point moving average to aid visual interpretation of trends. Deviations in monthly rainfall were also examined by comparing monthly values of these parameters obtained during the study period with synoptic mean values computed from the 8.25 years of surveillance. The disparity between long term values and current values was referred to as an anomaly and had either positive or negative values.

A regression model expressing malaria cases as a linear combination of non-malaria cases and rainfall was used to test for trend. The non-malaria cases and rainfall allow trend statistics to be presented that are "aware" of other potential longitudinal influences on malaria admissions. A continuous variable indicating the time in months from the start of the observation period was included in the regression model. The coefficient of time in the model estimates the trend in the series (i.e. the month-to-month change in the number of admission cases). A P-value of < 0.05 was considered significant. There were strong seasonal fluctuations observed for malaria admissions, with a higher proportion of admissions recorded in the rainy season than in the dry season. To control for the confounding effect of seasonality on the trend, seasonal effects were included in the regression model by the use of indicator terms (dummy variables). The 11 dummy variables are constructed as time series with the value one for observations falling in a given month and zero when not. The final month was used as the baseline for comparison. There are thus 11 potential intercepts generated depending on the choice of baseline month for comparison; we elected to plot and discuss the intercept and trend that had the maximum correlation with the 13-point moving average. These analyses were conducted separately for each district.

A key assumption when using ordinary least squares regression is that the model residuals are independent [18] and this is often violated by longitudinal data [19]. In the presence of autocorrelation standard errors of parameter estimates are underestimated resulting in an overestimation of significance. The Durbin-Watson statistic was hence used to test for serial autocorrelation [20]. Serial correlation was found in all malaria admission data series (results not shown) indicating that correction was necessary. An analysis of autocorrelation and the sample partial autocorrelation function of deseasonalised data [19,21] showed that a correction of this serial correlation with a lag of two months was optimal. Newey-West standard errors [21,22] with a lag of two months were therefore used to correct for autocorrelation and potential heteroscedasticity.

## Results

### Descriptions of the data

Longitudinal data was obtained from all the hospitals for the period starting January 1999 to March 2007. During the 8.25 years of surveillance there were a total of 76,101 paediatric admissions: 41,715 admissions in Kilifi, 13,492 in Kwale and 20,894 admissions in Malindi. A total of 34% of the total admissions had a diagnosis of malaria; 32% (13,919) in Kilifi, 40% (5,361) in Kwale, and 36% (7,498) in Malindi.

The annual number of malaria admissions declined with time (Table 1) in all three locations. In 1999 the proportion of admissions due to malaria was 46% in Kilifi, 51% in Kwale and 45% in Malindi. In 2006, seven years after the start of surveillance, this proportion had declined to 13% in Kilifi, 26% in Kwale and 24% in Malindi. The proportional decline was greatest in Kilifi (-72%) and lowest in Malindi (-38%). Conversely, within the same period, the proportion of admissions diagnosed as non-malaria increased (Table 1). The largest proportional increase in non-malaria cases was observed in Malindi at 64% while the lowest increase was recorded in Kwale 15%. The proportional increase in non-malaria admissions in Kilifi was 52%.

**Table 1:** Trends in paediatric admissions during the period January1999 to March 2007 at three sites on the Kenyan coast.

	***1999***	***2006***	*Change (%)*	*Intercept (95% confidence interval)*	*Trend (95% confidence interval)*
**Malaria Admissions**					
Total across sites	4611	1796	-61		
Kilifi	2395	660	-72	259.57 (155.64, 363.49)	-1.67^† ^(-2.29, -1.05)
Kwale	1053	418	-60	80.12 (58.88, 101.35)	-0.43^† ^(-0.65, -0.21)
Malindi	1163	718	-38	119.15 (84.00, 154.31)	-0.33^† ^(-0.59, -0.08)
					
**Non-Malaria Admissions**					
Total across sites	5215	7724	48		
Kilifi	2791	4243	52	236.72 (194.95, 278.50)	1.37^† ^(0.92,1.81)
Kwale	1022	1175	15	73.47 (50.16, 96.78)	-0.01^‡ ^(-0.31, 0.29)
Malindi	1402	2306	64	79.45 (57.55, 101.35)	0.91^† ^(0.60, 1.23)
					
**Rainfall**					
Kilifi	1197.7	2099.7	75	81.98 (38.27, 125.58)	0.10^‡ ^(-0.65, 0.85)
Kwale	1591.1	1910.2	20	48.96 (4.55, 93.37)	-0.04^‡ ^(-0.89, 0.80)
Malindi	1020.7	1505.2	47	84.32 (52.82, 115.83)	0.02^‡ ^(-0.40, 0.44)

^†^p < 0.001. ^‡ ^Not significant (p > 0.05).

### Seasonally adjusted linear regression analysis of admissions

**Figure 2:**
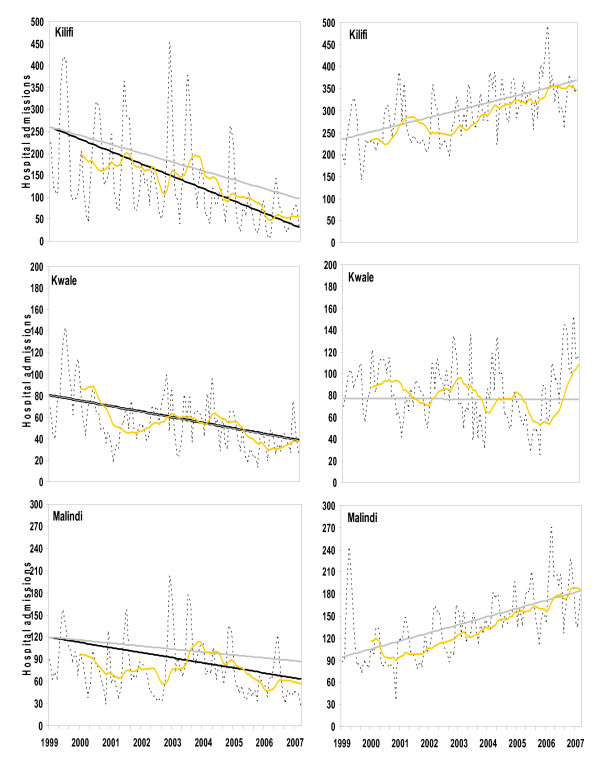
Admissions by month for the period January 1999 to March 2007 at three sites on the Kenyan coast. The top row is Kilifi, the middle row Kwale and the bottom Malindi. The graphs show malaria admissions (left column) and non-malaria admissions (right column) as dashed lines. The yellow solid line is a 13-point moving average applied to filter seasonal variation and highlight the long-term movements in the data. The two solid tone lines illustrate the change in admissions adjusted for seasonality (light grey) and seasonality, rainfall and non-malaria admissions (black). The intercept was chosen (from the potential 11) based on the maximal correlation with the 13-point m.a.

Malaria admissions in all three study sites were observed to show significant downward trends (Table 1). When adjusting for the increases in non-malaria admissions the decreasing trend in malaria admissions became more pronounced in Kilifi and Malindi (Table 2; P < 0.001). By March 2007 the estimated proportional decline in malaria cases was 63% in Kilifi, 53% in Kwale and 28% in Malindi. Values of the intercepts and coefficients of trends are detailed in Table 1 and shown in Figure 2 – left panel. Including rainfall as an additional covariate had little effect on the intercept and slope of the trends observed so that the trends were indistinguishable on Figure 2 (results not shown).

**Table 2:** Trend of monthly malaria admissions during period the period January 1999 to March 2007 at three sites on the Kenyan coast.

***Parameters***	***coefficient***	NW SE*	***t-stat***	***P-value***	***95% confidence interval***	
**Kilifi**						
Intercept	145.887	71.50	2.04	0.044	3.69	288.08
Trend	-2.320	0.40	-5.80	0.000	-3.12	-1.52
Non-mal cases	0.478	0.19	2.47	0.016	0.09	0.86
Rainfall	0.094	0.05	1.92	0.058	0.00	0.19
						
**Kwale**						
Intercept	52.933	12.62	4.20	0.000	27.84	78.02
Trend	-0.425	0.10	-4.19	0.000	-0.63	-0.22
Non-mal cases	0.377	0.08	4.64	0.000	0.22	0.54
Rainfall	-0.018	0.02	-0.78	0.435	-0.06	0.03
						
**Malindi**						
Intercept	95.395	19.54	4.88	0.000	56.53	134.26
Trend	-0.574	0.15	-3.78	0.000	-0.88	-0.27
Non-mal cases	0.262	0.14	1.84	0.069	-0.02	0.55
Rainfall	0.007	0.07	0.11	0.910	-0.12	0.14

*NWSE: Newey-West standard errors.

Significant (P < 0.001) upward trends in non-malaria admission cases were shown in both the Kilifi and Malindi hospitals over the study period (Table 1). There was no statistically significant difference in Kwale. By March 2007, non-malaria admission cases in Kilifi and Malindi were estimated to have increased by 56% and over 100% respectively (Figure 2 – right panel) while remaining relatively consistent in Kwale. Again including rainfall as an additional covariate had little effect on the intercept and slope of the trends observed (results not shown).

### Seasonally adjusted linear regression analysis of rainfall and anomalies

**Figure 3:**
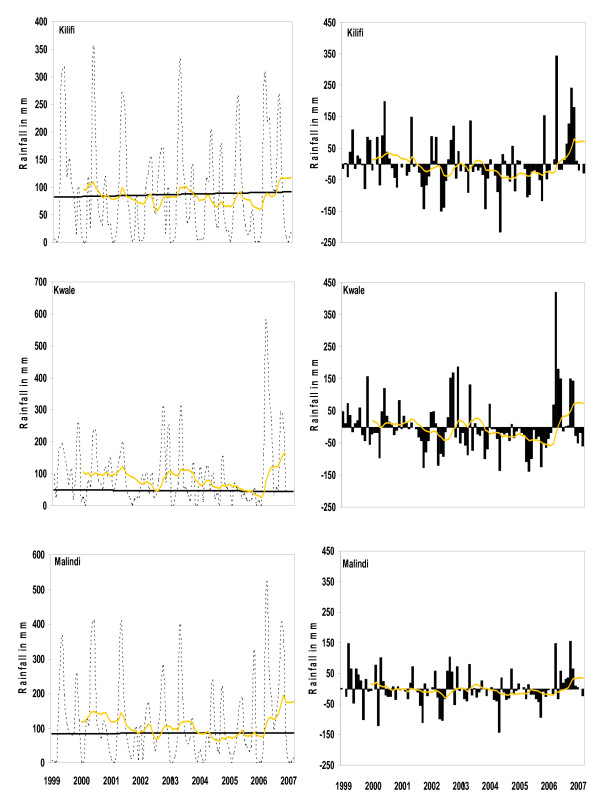
Rainfall per month for the period January 1999 to March 2007 at three sites on the Kenyan coast. The top row is Kilifi, the middle row Kwale and the bottom Malindi. The graphs in the left column show the monthly rainfall in mm as dashed lines. The yellow solid line is a 13-point moving average applied to filter seasonal variation and highlight the long-term movements in the data. Trends corrected for seasonality are shown in black. The rainfall expressed as anomalies relative to 1999–2007 monthly mean for each site are shown in the middle column. The 13-point moving average is again also shown. The intercept was chosen (from the potential 11) based on the maximal correlation with the 13-point m.a.

The trend in rainfall patterns remained relatively stable during the 8.25 years of surveillance. There was no evidence of consistent changes in rainfall patterns over the period with no significant synoptic trends identified in any of the three study sites (Table 1, Figure 3 – left panel). The anomaly analyses show that during the period 2004–2005, monthly rainfall in all the three study sites was lower on average than the computed long term means (Figure 3 – right panel). Thus there were no notably drier years except during this period. This was followed by considerable increase in rainfall in 2006 in all the study sites.

## Discussion

Between January 1999 and March 2007 (8.25 years) admissions due to malaria have systematically declined at three sites along the Kenyan coast. These declines were examined in relation to possible changes in overall hospital utilization by a naturally growing paediatric population size and within and between year variations in rainfall. The fact that the significant decline in malaria admissions occurred against a significant rise in non-malaria admissions in Kilifi and Malindi and remained unchanged at Kwale during the surveillance period (Figure 1, right panel) suggests that the declining admissions due to malaria were specific to malaria. It could be argued that an increase in non-malaria cases might be attributed to a changing diagnostic pattern over the surveillance period with fewer misclassifications or more doctors accepting as true malaria test results. This cannot be ruled out at Malindi or Kwale but at Kilifi standardized diagnostic practices have applied to all paediatric admissions throughout the entire surveillance period and thus changes in the pattern of diagnosis seem an unlikely explanation for the trends observed.

The monthly incidence of malaria is coupled to seasonal rainfall patterns on the Kenyan coast. The addition of rainfall as a covariate to the model had no effect on the slope or intercept of seasonally corrected trend lines because rainfall patterns were shown to have remained relatively constant during the observation period with no significant increasing or decreasing trends observed in any of the study sites. Attributing the long-term reduction in malaria cases to a constant change in rainfall is implausible as there was no evidence of a substantial decline in rainfall except for the anomaly recorded in 2004/5, where rainfall was lower on average than that expected (Figure 3, right panel). This was however followed by a considerable substantial increase in amount of rainfall recorded in 2006. Despite this there was no associated increase in malaria admissions observed in 2006 or early 2007.

The use of three different hospital admission series increases the external validity of a single observation from one hospital. We consider the data from Kilifi as the diagnostic gold standard based on the use of microscopy and a discharge diagnosis supported by review of clinical notes. Using this as our point of reference, external validity of this result is provided by data from Msambweni (Kwale) and Malindi. It is reassuring then that a consistent declining pattern in malaria admission is maintained across all study sites lending weight to the strength of the findings. By 2006, annual malaria admissions had decreased by an average of 57% compared to 1999 across all sites.

**Figure 4:**
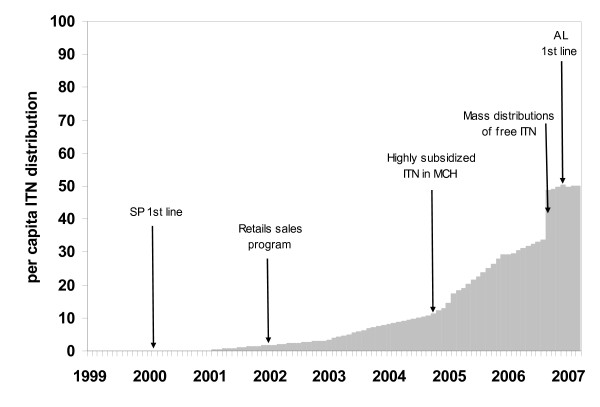
Cumulative monthly ITN distribution volumes expressed per capita across the three districts of Malindi, Kilifi and Kwale.

There are several possible factors that might explain these observations and are considered as plausibility arguments, as proposed by Habicht [23] and Victora [24], rather than measurable correlates. The most notable programmatic change over the 8.25 years of surveillance has been the increasing use of ITNs [4]. We have assembled a population adjusted estimate of the per capita ITN distribution patterns between 1999 and 2007 across the three districts combined (Figure 4). At the start of the observation period ITN distribution in all the three study sites was negligible. During the period 2001–2004, there was a steady increase in the cumulative per capita ITN distribution (Figure 4). One year after initiation of a retail sector programme (beginning of 2003), cumulative ITN distribution was estimated to be 3% per capita across the three districts. By December 2004, three months after the inception of an MCH clinic sales programme, cumulative ITN distribution was over 13 nets per 100 people. The largest increase occurred in September 2006 during the mass distribution campaign from an estimated 0.34 nets per capita in August 2006 to 0.49 nets per capita by September 2006. Extending the surveillance from 2006 into March 2007 corresponds to a period of highest ITN "coverage", implementation of a new effective first line treatment policy and increasing rainfall. Even so the anomaly in malaria admissions recorded was greatest during this period with an average of -79 in Kilifi, -34 in Malindi and -14 in Kwale. It should, however, be recognized that the decrease in malaria cases started before the major expansion of prevention coverage in 2006, reasons for this remain unclear.

Data on the serial use of prompt, effective antimalarial treatment in each of the three districts was not possible to assemble. However a semi-qualitative, temporal description of drug efficacy is possible in accordance with national first line recommendations for treatment between 1999 and 2007. Between January 1999 and early 2000 chloroquine was still the only available antimalarial in government clinics despite wide-spread resistance documented on the coast [25]. Between mid-2000 and April 2006 sulphadoxine-pyrimethamine (SP) was the nationally recommended first line drug available in most government clinics and drug efficacy declined rapidly over this period in Kilifi and Kwale until most studies showed at least 25% failure rates when undertaken after 2002 [26,27]. The policy was changed in April 2004 to artemether-lumefathrine (AL) but was not effectively implemented until December 2006 [5]. The efficacy of AL was defined in Kilifi as in excess of 94% in 2002–2003 [28]. Patient access to antimalarials within 48 hours of onset of symptoms has been documented in Kwale district in 2001 (15%) and 2006 (17%) ([29]; unpublished data), both representing very low estimates of prompt access and we assume not dissimilar to anticipated access figures for Kilifi and Malindi districts during the same period. However, the widespread availability of even sub-efficacious SP over-the-counter from 2000 onwards in Kwale [30,31] and Kilifi [32] may have had a suppressive effect on clinical disease risks operating similar to strategies prompting intermittent presumptive treatment in young children [33].

The use of hospital data provides a useful indicator of the long-term and short-term impact of scaling-up malaria interventions. It is not possible to definitely attribute carefully controlled and adjusted changes in malaria admission rates to expanded coverage of preventative and curative interventions; however these seem to be the most parsimonious explanations for the observations reported here along the Kenyan coast.

## Authors' contributions

EA Okiro assembled all the hospital data, developed the analytical models and wrote the manuscript; SI Hay provided technical support for the time-series models and contributed to the drafting of the manuscript; PW Gikandi supervised the collection of all the hospital data since 1999; SK Sharif was the Provincial Medical Officer for Coast province between 1999 and 2005 responsible for the delivery of services and collection of health information and contributed to the final draft of the manuscript; AM Noor was responsible for the assembly of the ITN data in each district and contributed the final manuscript; N Peshu and K Marsh were overall responsible for the data provided by Kilifi district hospital and contributed to earlier drafts of the manuscript. RW Snow was responsible for the conception and continued funding of the project and its overall scientific management, analysis, interpretation and preparation of the final manuscript. All authors read and approved the final manuscript.

## Appendix

Table 1

**Footnote**: The intercept refers to the seasonally adjusted level of malaria and non-malaria cases and rainfall at the start of the observation period. Models for malaria and non-malaria included a covariate for rainfall. The regressions were performed with Newey-West standard errors with a lag of two months for models with malaria and non-malaria case outcomes and a lag of one month for models with rainfall.

Table 2

**Footnote**: The intercept refers to the seasonally adjusted level of malaria cases at the start of the observation period. Models included a covariate for non-malaria cases and rainfall. The regression was performed with Newey-West standard errors with a lag of two months for models with malaria and non-malaria case outcomes.

Figure 4

**Footnote**: Information on net deliveries was assembled from a variety of sources for each district. We assumed that ITN coverage was less than 5% between January 1999 and December 2002. This position is supported by reviews of net use undertaken in Kenya during this period [34]. In Kilifi district a large scale trial of ITN was completed in 1993 [35] and by 1997 there were few net replacements or net re-treatments [36]. From 2002 Population Services International (PSI) had launched a retail sector, social marketing campaign for ITN distribution [37]. Annual sales figures for the coast were obtained from PSI for the period January 2002 through to December 2004. Toward the end of 2004 PSI delivered heavily subsidized ITN through clinics and monthly sales by geo-positioned clinics in each of the three districts were available for the period November 2004 to March 2007. In September 2006 the Ministry of Health launched a large-scale free distribution campaign of free ITN to children under the age of five years [4]. Net distribution volumes were recorded per geo-located distribution point within each district. Finally, small-scale community distribution projects over the surveillance by the district heath management team, NGO's and philanthropic organizations were recorded through interviews with DHMT members and district stakeholders and recorded as volumes of distribution and month of delivery. Cumulative monthly ITN distribution volumes per district were computed per capita using population size estimates derived from national census data and annual growth rates. This was implemented with projected population growth rate curves from national inter-censal district-specific annual rates of net population increase derived in 1989 and 1999 [38].
